# Comparison of the Effectiveness of Grape Seed Extract and Low‐Level Laser Therapy in the Management of Dentin Hypersensitivity: A Randomized Controlled Trial

**DOI:** 10.1155/sci5/6656174

**Published:** 2026-02-12

**Authors:** Tun Yi Darren Ong, Wei Nee Lim, Li Jia Ong, Ju Wen Lim, Gursimrendeep Kaur, Anchu Rachel Thomas, Venkata Bharatwaj Narasu

**Affiliations:** ^1^ Department of Conservative Dentistry and Endodontics, Manipal University College Malaysia, Melaka, Malaysia; ^2^ Department of Conservative Dentistry and Endodontics, Faculty of Dentistry, Manipal University College Malaysia, Melaka, Malaysia; ^3^ Department of Periodontics, Faculty of Dentistry, Manipal University College Malaysia, Melaka, Malaysia

**Keywords:** air spray, cold test, dentin hypersensitivity, grape seed extract, low-level laser therapy, visual analog scale (VAS)

## Abstract

**Objective:**

To evaluate the effectiveness of grape seed extract (GSE) and its combination with low‐level laser therapy (LLLT) in alleviating pain from dentin hypersensitivity (DH) compared to LLLT alone.

**Methods:**

Patients aged 20–50 years with at least three sensitive teeth in different quadrants and baseline pain scores ≥ 2 on the visual analog scale (VAS) were recruited. Each tooth was randomly allocated to one of three groups: LLLT + placebo, GSE + placebo, or GSE + LLLT. Pain scores were recorded on Days 1, 3, and 7 using cold and air spray tests.

**Results:**

The GSE + LLLT group demonstrated the greatest reduction in pain sensitivity, showing a 72.8% decrease for air spray and 59.51% for the cold test at Day 3, compared to the LLLT or GSE groups alone. However, there were no statistically significant differences (*P* > 0.05) in pain score reduction among the groups at any time point.

**Discussion:**

The combination of GSE and LLLT may enhance early pain reduction in DH compared to individual treatments, but the improvement was not statistically significant. GSE presents a potentially feasible and patient‐friendly alternative for DH management, warranting further clinical validation.

**Statistical Analysis Used:**

Data were collected and analyzed using IBM Statistical Package for the Social Sciences (SPSS) software program, Version 20.0 (IBM, Chicago, Illinois). General linear model‐repeated measure ANOVA was carried out to assess the reduction in DH pain score among individual interventions and the comparison between the three interventions. The significance level was set at *p* < 0.05.

**Conclusion:**

While GSE combined with LLLT showed the greatest numerical reduction in DH pain scores, the difference was not statistically significant. Further large‐scale studies are recommended to confirm the clinical benefits of this combination.

**Trial Registration:**

ClinicalTrials.gov identifier: NCT05927831

## 1. Introduction

Dentin hypersensitivity (DH) is described as a sharp, transient pain originating from exposed dentin in response to normally innocuous stimuli, including temperature changes, evaporative stimuli, touch, changes in osmotic pressure, or certain chemicals [1]. It presents treatment challenges, impacts oral health–related quality of life, and interferes with routine activities like speaking, eating, and brushing teeth [2, 3]. Pain is not linked to other dental conditions, with the prevalence in adults varying widely from 1.3% to 92.1% [4].

DH arises from exposed dentinal tubules due to tooth wear, periodontal tissue loss, gingival recession, or developmental lesions [5]. The exposed tubules connect the oral environment to the pulp, allowing stimuli to reach nerves [6]. Research indicates that the dentinal tubules in hypersensitive teeth (0.83 μm) are nearly twice as wide as those in normal teeth (0.4 μm), increasing fluid movement and pain sensation. This heightened reactivity leads to hypersensitivity [7, 8].

Current management strategies for DH include various approaches including nerve desensitization, the application of anti‐inflammatory agents, and occlusion of exposed dentinal tubules [6]. The primary goal of DH treatment is to provide sustained relief by creating a physical barrier over exposed dentin, which blocks hydrodynamic fluid movement in dentinal tubules, reducing nociceptor stimulation and pain [9].

Owing to its multifaceted effects, low‐level laser therapy (LLLT) has emerged as a promising treatment for DH. LLLT exerts analgesic, biostimulatory, and anti‐inflammatory properties within the dentin–pulp complex [10]. It has been reported to be able to alter the neuronal physiology of sensory nerves and control microinflammation within the dentin and pulp. These combined actions, including potential regulation of cellular metabolism, contribute to the effectiveness of LLLT in alleviating DH [11, 12]. LLLT may be used in various wavelengths; however, 810‐nm LLLT was found more effective than 660 nm, providing long‐lasting relief for DH [12].

In recent times, research on naturally derived ingredients as medicinal agents has increased. Grape seed extract (GSE) (*Vitis vinifera*) is known for being rich in nutrients and medical properties. GSE is reported to have anti‐inflammatory, antimicrobial, and antioxidant properties [13] It contains proanthocyanidins (PAs), which interact with proline‐rich proteins such as collagen and support the activity of proline hydroxylase, an enzyme crucial for collagen synthesis in dentin [14, 15], aiding in remineralization. The cross‐linking action of collagen fibrils in dentin can prevent mineral loss and increase dentin mechanical properties as well as the stability of matrix [16]. In vitro studies conducted by Mirkarimi et al. and Olmez et al. revealed the formation of cluster‐like deposits on the enamel surface treated with GSE. The deposits are hypothesized to be calcium fluoride, potentially attributable to the fluoride content present in GSE [17, 18].

A previous study reported synergistic effect between LLLT and sodium fluoride gel in managing DH [8]. Compared with LLLT alone (either at 650 nm or 810 nm wavelength), the combination therapy demonstrated superior pain reduction efficacy [8]. This finding suggests that combining LLLT with other agents might be a promising strategy for enhancing DH control. There is limited research evaluating the effectiveness of GSE in managing DH, particularly in comparison to established treatments like LLLT. Therefore, this study aimed to evaluate the effectiveness of GSE and the combination of GSE with LLLT in the reduction of pain related to DH in comparison with the use of LLLT alone. The null hypothesis states that there are no differences in the reduction of the pain score of patients with DH following the use of LLLT, GSE, or the combination of GSE with LLLT.

## 2. Materials and Methods

Ethical approval for the study was granted by the Institutional Research and Ethics Committee (MUCM/Research and Ethics Committee‐018/2023), and the study was registered with ClinicalTrials.gov. All procedures were conducted in compliance with applicable laws and institutional regulations. The study was conducted in accordance with the Helsinki Declaration revised version 2013. The manuscript has been written according to the CONSORT 2010 guidelines [19].

A split‐mouth, double‐blinded randomized clinical trial was conducted. Informed consent was obtained from all individual participants included in the study. The participants received a comprehensive explanation of the study’s objectives, procedures, potential risks, and benefits prior to providing consent for the trial.

**Figure 1:**
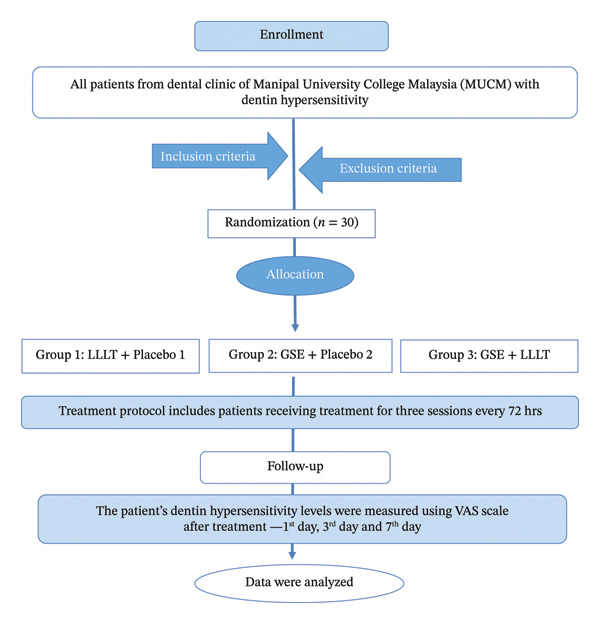
Flowchart—enrollment of participants.

Patients with DH between 18 and 50 years of age visiting the MUCM dental clinic following the inclusion criteria were selected. Patients who voluntarily provide written informed consent to participate in the study with at least three sensitive teeth in three different quadrants either due to gingival recession: score < 2 based on Miller’s classification [20], patients with a Tooth Wear Index (TWI) score of less than 3, as per the Smith and Knight TWI [21], patients classified as ASA Physical Status I or II according to the American Society of Anesthesiologists classification [22] and presenting with a visual analog scale (VAS) pain score of ≥ 2, were included in the study. Teeth with caries, cracked or fractured teeth, mobile teeth, and pulpitis were excluded. As part of the periodontal assessment, a comprehensive periodontal examination, including probing depth and gingival recession evaluation, was performed. Teeth with probing depths > 6 mm or a history of periodontal surgery within the preceding 3 months were excluded from the trial. Additionally, patients who had undergone recent desensitizing therapy, those with systemic diseases, individuals with known allergies to the active ingredients, and patients with gastric disorders (GERD) were excluded (Figure 1).

The sample size calculation was based on the study by Pandey et al. [10]. Using G^∗^Power 3.1, a sample size of 32 participants was determined to achieve 90% power, with an effect size of 0.64 and significance level of 0.05. Randomization was done using randomizer.org and Microsoft Excel. Block randomization was performed using block size of 6. Each quadrant was assigned randomly to a treatment; allocation concealment was done by inserting each combination in an envelope, with the participant’s sample number attached to it. The patient and the assessor were blinded. The GSE and the placebo were stored in opaque colored bottles. The random allocation sequence and enrollment of participants were carried out by different people coordinating the trial.

The teeth undergoing the interventions were isolated using rubber dam (Coltene, Coltene Malaysia, Petaling Jaya). For Group 1, LLLT + placebo: The LLLT device (Gemini, Ultradent, Selangor) was utilized at wavelength of 810 nm and a power output of 0.5 watts, with a tip diameter of 300 μm. The application began 5‐6 mm from the hypersensitive tooth area and gradually moved closer to a range of 2‐3 mm. A scanning motion was employed to cover the entire hypersensitive region, with the duration of exposure ranging from 30 s to 1 min [10]. Prior to LLLT application, the placebo was administered (glycerine + yellow food color) topically using an applicator brush to the hypersensitive area and retained for 10 min [23].

Group 2, GSE + placebo: The GSE extract (*Vitis vinifera*, standard 95% [23.75 mg] PAs, Natures Plus, Herbal Actives, Melville, New York) (Product code: NAP‐07832) (Table 1) was applied topically to the hypersensitive area and retained for 10 min. The patients were given colored protective eye wear, and the placebo was achieved by placing the LLLT on the tooth surface without switching on the device (fake LLLT).

**Table 1:** Ingredients—grape seed extract.

Category	Ingredient
Active ingredient	Grape seed (*Vitis vinifera*) extract—25 mg (standardized to 95% proanthocyanidins, ∼23.75 mg)
Other ingredients	Vegetable glycerin, purified water, potassium sorbate (preservative)

*Note:* The grape seed (*Vitis vinifera*) extract dose of 25 mg corresponds to approximately 23.75 mg of active proanthocyanidins, as the extract is standardized to 95% proanthocyanidin content.

In Group 3, GSE + LLLT: The GSE was applied topically to the hypersensitive area, followed by LLLT being performed as described above.

The VAS scores were recorded for each group at different time intervals at baseline, first day, third day, and seventh day ranging from 0 to 10 using both air spray and cold test, with a 5‐min interval between each stimulus. The air spray test was carried out by using the three‐way syringe attached to the dental chair, kept at 0.5 cm, perpendicular to the tooth surface for 1 s per tooth at room temperature. For the cold test, Endo Ice (Coltene, Coltene Malaysia, Petaling Jaya) was sprayed onto a Q tip (Ultradent, Selangor) and placed on the tooth surface for 5–10 s, and the score was recorded. The scores for the air spray test and cold test were recorded separately. Colored protective eyewear was provided to patients during the administration of all interventions.

Following the treatment, oral hygiene instructions were given to the patients. Adherence to a nonacidic diet was recommended. Consumption of fizzy drinks and alcoholic beverages was discouraged. Gentle brushing with an extra‐soft or ultra‐soft toothbrush was advised for 40 minutes after meals using the modified Stillman technique. Patients exhibiting parafunctional habits or bruxism were instructed to wear their nightguards consistently [10, 12].

### 2.1. Statistical Analysis

Data were collected and analyzed using IBM Statistical Package for the Social Sciences (SPSS) software program, Version 20.0 (IBM, Chicago, Illinois). General linear model‐repeated measure ANOVA was carried out to assess the reduction in DH pain score among individual interventions and the comparison between the three interventions. The significance level was set at *p* < 0.05.

## 3. Results

**Figure 2:**
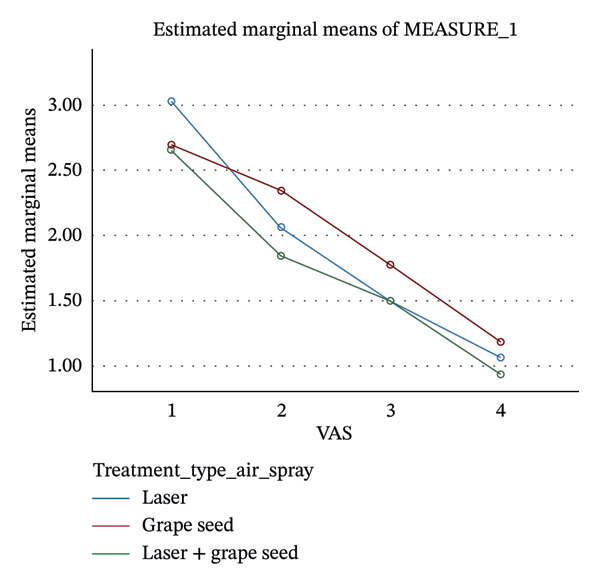
Reduction in pain scores following the use of the three interventions assessed with air spray. Note: MEASURE_1 refers to pain score measured using the Visual Analog Scale (VAS).

**Figure 3:**
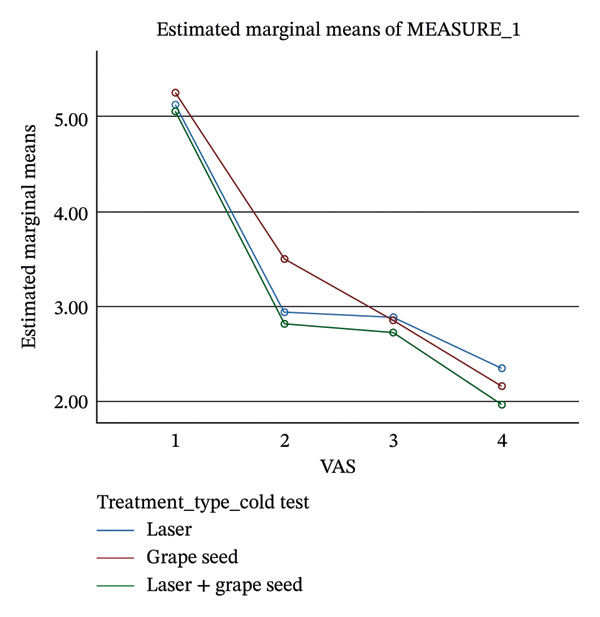
Reduction in pain scores following the use of three interventions assessed with cold test. Note: MEASURE_1 refers to pain score measured using the Visual Analog Scale (VAS).

Thirty patients (25 females and 5 males) with a mean age of 21–30 years were evaluated for 7 days (Table 2). There was overall reduction in DH pain scores in the three groups individually from Day 1, Day 3, and Day 7 (*P* < 0.0001). For the comparison of efficacy of LLLT, GSE, and their combination in the reduction of DH, pain scores were assessed from baseline to Day 7. The LLLT + GSE group demonstrated maximum reduction in pain sensitivity to air spray (72.8%) and cold test (59.51%) at Day 3 compared to LLLT or GSE alone (Table 3) (Figures 2 and 3). However, no statistically significant differences (*P* > 0.05) in pain score reduction were observed among the three groups at Days 1, 3, and 7 (Table 4).

**Table 2:** Sociodemographic profile.

Demographics	Description	Number	Percentage
Age	20 and below	0	0%
	21–30	28	87.5%
	31–40	4	12.5%
	41–50	0	0%
	Total	32	100%
Gender	Male	7	21.875%
	Female	25	78.125%

**Table 3:** Percentage reduction in pain score.

Day	Mean (standard deviation) (air spray)	Percentage reduction	Mean (standard deviation) (cold test)	Percentage reduction
3a. Reduction in pain score: Group I: LLLT				
Baseline	3.03 (1.94)	—	5.13 (2.01)	—
1	2.06 (2.06)	38.79%	2.94 (2.29)	43.3%
3	1.50 (1.52)	53.84%	2.88 (1.91)	42.7%
7	1.06 (1.46)	67.53%	2.34 (1.98)	51.5%
3b. Reduction in pain score: Group II: GSE				
Baseline	2.69 (2.32)	—	5.25 (2.06)	—
1	2.34 (2.25)	30.19%	3.50 (2.57)	38.37%
3	1.78 (1.75)	47.27%	2.84 (2.13)	48.54%
7	1.19 (1.62)	64.85%	2.16 (2.14)	59%
3c. Reduction in pain score: Group III: LLLT + GSE				
Baseline	2.66 (2.15)	—	5.06 (2.29)	—
1	1.84 (1.89)	47.09%	2.81 (2.36)	46.5%
3	1.50 (1.70)	52.65%	2.72 (2.57)	52.09%
7	0.94 (1.37)	72.87%	1.97 (1.77)	59.51%

**Table 4:** A comparison among LLLT, GSE, and combination of both LLLT and GSE using air spray and cold test.

Evaluation of pain score with air spray	Air spray (*p* value)	Cold test (*p* value)
Within‐subject comparison (baseline, Day 1, Day 3, Day 7)	< 0.001^∗∗∗^	< 0.001^∗∗∗^
Between‐subject comparison (LLLT, GSE, LLLT + GSE)	0.839	0.813

*Note:* Repeated measures ANOVA.

^∗∗∗^ *p* < 0.001 (statistically highly significant); repeated measures ANOVA.

## 4. Discussion

This study aimed to compare the efficacy of LLLT, GSE, and their combination in managing DH. Pain scores were assessed from baseline to Day 7 to evaluate the effectiveness of each intervention. A split‐mouth, double‐blinded study design was carried out to facilitate the comparison of the three treatment methods under similar and standardized conditions.

Based on the data collected in this study regarding the percentage reduction in DH pain scores for each group, LLLT (Group I) demonstrated a significant decrease in pain by Day 7 compared to the baseline, Day 1, and Day 3. This finding aligns with the studies by Pandey et al. and Praveen et al., who reported notable reductions in pain scores after three LLLT applications. These results can be attributed to LLLT’s biomodulatory, analgesic, and anti‐inflammatory effects [10, 24]. Additionally, its ability to modulate neural transmission plays a key role in alleviating DH‐related pain. Specifically, LLLT induces temporary distension in axons of nerves, resulting in a reversible blockade of action potentials [25]. This neuromodulatory effect, combined with LLLT’s capacity to stimulate normal physiological processes, contributes to the lasting pain relief observed 1 week after treatment.

GSE (Group II) also showed a significant reduction in pain scores at Day 7 from baseline, Day 1, and Day 3, suggesting its potential as a viable alternative treatment for DH. The application of GSE to the tooth surface for 10–20 s, followed by a 10‐minute retention period, was based on the findings in the in vitro study by Saragih et al. [23], which utilized a gel‐based formulation. In contrast, the present study employed a water‐based GSE, maintaining the same application duration. This variation in formulation likely enhanced the extract’s penetration and antibacterial efficacy, as the hydrophilic nature of dentinal tubules may have facilitated more rapid and effective diffusion. This protocol aimed to optimize GSE penetration and its potential for enhancing enamel hardness by mineral deposition and remineralization [26–28].

The LLLT + GSE group (Group III) showed the highest reduction in pain scores, particularly in response to air spray and cold tests, on Day 7 compared to Groups I and II. However, there were no statistically significant differences among the three groups on Days 1, 3, or 7, indicating the need for further research. The observed effects may result from the combined interaction between LLLT and GSE, where LLLT’s biostimulatory pain‐reduction effect combines with GSE’s remineralizing properties to enhance the overall therapeutic outcome. This compatibility could have facilitated the absorption of the GSE into the tubules, potentially enhancing its therapeutic effects in reducing DH. Previous studies on DH pain reduction have suggested that combining LLLT with other agents is effective [10, 29].

In addition to comparing laser‐based protocols, it is necessary to consider the current results in relation to combination with topical desensitizing systems. Conventional topical treatments, such as potassium nitrate, typically alleviate sensitivity by desensitizing neural function [29]; conversely, fluoride varnishes work by blocking dentin tubules and encouraging mineral deposition [24]. The effectiveness of these treatments can vary based on the duration of observation and the type of stimulus. In a clinical trial conducted by Pandey and colleagues, 5% potassium nitrate treatment, LLLT, and their combination decreased DH [10]. However, the combined approach demonstrated a more favorable clinical outcome compared to either treatment alone, suggesting that combining photobiomodulation with a topical desensitizer could be beneficial in reducing the symptoms. In a randomized split‐mouth trial, Jain et al. evaluated 5% sodium fluoride varnish, 810‐nm diode laser, and their combination, reporting significant improvement across groups over time, with laser‐containing protocols demonstrating greater reductions than varnish alone, and no consistent superiority of the combined protocol over laser monotherapy at longer follow‐ups [29]. These reports suggest that the clinical advantage of combination therapy may be dependent on the agent, delivery mode, number of sessions, baseline severity, and duration of follow‐up.

Glycerine was used as a placebo for the GSE to ensure consistency of the vehicle, as GSE is glycerine‐based. Although a study conducted in 1990 [30] suggested that glycerine might influence DH, it lacks GSE’s remineralizing properties, and its mechanism remains unexplained in the study. Any minor effect of glycerine would be the same in both groups, ensuring that any observed differences are solely due to the bioactive components of GSE.

An area for improvement in this study could be the inclusion of a control group receiving a placebo and fake (sham) LLLT irradiation, which would strengthen the study’s validity and allow for more robust comparisons.

This study provides preliminary evidence suggesting that the application of GSE may be a promising approach for managing DH. While the combination exhibited superior pain reduction, further trials with larger patient participation along with equal distribution of age and gender and extended follow‐up periods are necessary to confirm the long‐term efficacy and generalizability of these findings.

## 5. Conclusion

GSE and LLLT were both effective in reducing DH over a 7‐day period. Although the combination of GSE and LLLT demonstrated the greatest reduction in pain sensitivity, particularly to air spray and cold stimuli by Day 3, the differences among the three groups were not statistically significant. Nevertheless, the clinical trend suggests potential additive benefits when both modalities are used together. GSE, as a home‐based treatment option, offers convenience and accessibility, making it a promising alternative for patients seeking relief from DH.

## Author Contributions

Conceptualization, Anchu Rachel Thomas; methodology, Anchu Rachel Thomas, Tun Yi Darren Ong, and Wei Nee Lim; software, Tun Yi Darren Ong, Wei Nee Lim, Li Jia Ong, Ju Wen Lim, and Gursimrendeep Kaur; validation, Tun Yi Darren Ong, Wei Nee Lim, Li Jia Ong, Ju Wen Lim, Gursimrendeep Kaur and Anchu Rachel Thomas; formal analysis, Tun Yi Darren Ong, Wei Nee Lim, Li Jia Ong, and Venkata Bharatwaj Narasu; writing–original draft preparation, Tun Yi Darren Ong, Wei Nee Lim, Li Jia Ong, Ju Wen Lim, Gursimrendeep Kaur and Anchu Rachel Thomas; writing–review and editing, Anchu Rachel Thomas; supervision, Anchu Rachel Thomas and Venkata Bharatwaj Narasu; and project administration, Anchu Rachel Thomas.

## Funding

This study was funded by the Faculty of Dentistry, Manipal University College Malaysia, Melaka, Malaysia.

## Disclosure

All authors have read and agreed to the published version of the manuscript.

## Consent

Written informed consent was obtained from all individual participants included in the study.

## Conflicts of Interest

The authors declare no conflicts of interest.

## Data Availability

All relevant data are included in the article. The datasets used and/or analyzed during the current study are available from the corresponding author on reasonable request.
